# Behavioral Changes in Patients with Multiple Sclerosis

**DOI:** 10.3389/fneur.2017.00437

**Published:** 2017-08-28

**Authors:** Mirjam R. Heldner, Sigal Kaufmann-Ezra, Klemens Gutbrod, Corrado Bernasconi, Sandra Bigi, Verena Blatter, Heinrich P. Mattle, René M. Müri, Rajeev K. Verma, Christian P. Kamm

**Affiliations:** ^1^Department of Neurology, Inselspital, Bern University Hospital, University of Bern, Bern, Switzerland; ^2^University Neurorehabilitation, Department of Neurology, Inselspital, Bern University Hospital, University of Bern, Bern, Switzerland; ^3^Department of Neuropediatrics, Inselspital, Bern University Hospital, University of Bern, Bern, Switzerland; ^4^Institute of Diagnostic and Interventional Neuroradiology, Inselspital, Bern University Hospital, University of Bern, Bern, Switzerland; ^5^Neurology and Neurorehabilitation Center, Luzerner Kantonsspital, Luzern, Switzerland

**Keywords:** multiple sclerosis, cognition, behavioral changes, Frontal Systems Behavior Scale, disability evaluation

## Abstract

**Background:**

Behavioral changes are common in patients with multiple sclerosis (MS), however not as readily recognized as cognitive impairments.

**Objective:**

The aim of this study was to analyze behavioral changes and its relation to disease characteristics, disability, and cognitive impairments in patients with MS.

**Method:**

This is a single-center cross-sectional study. A detailed neuropsychological examination, including the Frontal Systems Behavior Scale (FrSBe), the Beck depression inventory (BDI), and the Wuerzburg Fatigue Inventory for Multiple Sclerosis (WEIMuS) test, was performed. FrSBe results were correlated with disease characteristics, disability, and cognitive assessments.

**Results:**

66 patients were enrolled (mean age: 43.4 years; disease duration: 9.3 years; Expanded Disability Status Scale: 3.0). Up to one third of patients showed behavioral changes in at least one domain or the total score of the FrSBe. Patients were mildly affected with regard to cognitive functioning. Consistent correlation was found between behavioral changes and fatigue (WEIMuS) and depressive symptoms (BDI), but not with disease characteristics, disability, or cognitive functions. There was an increase of behavioral changes on all FrSBe scales in the current status compared to the retrospectively rated status before disease onset. Self- and family ratings with regard to current behavioral changes were similar.

**Conclusion:**

Behavioral changes are common in otherwise mildly affected MS patients with up to one third being affected. In this patient cohort, behavioral changes occur largely independent of disease characteristics, physical disability, and cognitive functioning but correlate with both fatigue and depressive symptoms. Therefore, they should be tested specifically.

## Introduction

Multiple sclerosis (MS) is a chronic inflammatory disease of the central nervous system characterized by inflammation, demyelination, axonal injury, and axonal loss. MS affects gray and white matter of the whole brain and spinal cord eventually leading to diffuse gray and white matter atrophy (1).

Behavioral changes are common and manifold in MS with depressive symptoms being the most frequent reported neuropsychiatric manifestation with a lifetime prevalence rate of about 50% (2–4).

Other common neuropsychiatric manifestations include lability (41%), irritability (38%), inflexibility (26%), aggression (23%), impatience (22%), apathy (22%), adjustment disorder (17%), and obsessive-compulsive disorder (15%) (5). Behavioral changes are relevant because they negatively influence activities of daily living and contribute to poor quality of life and unemployment together with other symptoms such as physical disability and cognitive deficits (6–8). In addition, behavioral changes can be predictive of cognitive and functional impairment in MS patients (6, 9). Despite their frequency and relevance, behavioral changes receive little attention in the daily care of MS patients and in clinical trials. This is at least partly due to difficulties in assessing and quantifying behavioral changes.

In contrast, cognitive impairments can be evaluated and quantified more easily. In MS patients, cognitive impairments such as deficits in complex attention, efficiency of information processing, executive functioning, processing speed, and long-term memory are typical and noted early in the disease course (6, 9–12). The goal of the present study was to analyze behavioral changes in patients with MS with regard to its frequency and the patient population in whom behavioral changes can be predominantly expected. These are important information in the management of MS patients. To do so, the Frontal Systems Behavior Scale (FrSBe) that analyzes behavioral changes related to frontal lobe disturbances (apathy, disinhibition, and executive dysfunction) was correlated to MS-specific disease characteristics, disability, cognitive impairment, as well as depressive symptoms and fatigue (3, 6, 9, 12, 13).

Furthermore, we wanted to analyze the difference in asking patients and significant others in evaluating behavioral changes to gain information on the optimal way to evaluate behavioral changes in patients with MS in the daily practice and in clinical trials.

We hypothesized that behavioral changes (1) correlate with more severe disease stages with regard to general disability and disease duration, (2) correlate with cognitive impairments and especially with frontal lobe dysfunctions, (3) correlate with fatigue and depressive symptoms as shown in previous studies (3, 6, 9). Furthermore, we hypothesized that there is a difference in asking patients and significant others in rating behavioral changes as shown in previous studies in MS and in other brain diseases (13–17).

## Materials and Methods

### Patients

Consecutive patients with clinically isolated syndrome (CIS), relapsing-remitting MS, secondary progressive MS, or primary progressive MS (PPMS), according to the 2010 McDonald’s criteria, were recruited from our outpatient clinic (18). Main exclusion criteria were relapses and/or steroid treatment within the preceding 2 months and additional diseases or conditions apart from MS that affect behavioral changes or compromise the adequate performance of the study procedures.

### Study Design

This was a single-center, cross-sectional study. Patient characteristics were obtained from the last visit in the outpatient clinic including the Expanded Disability Status Scale (EDSS) that was performed by certified level C raters (http://neurostatus.net) (19).

All additional tests were performed in one extra session by a neuropsychologist (SK-E). Each patient provided written informed consent prior to study entry, and the study was approved by the ethics committee of the canton of Bern, Switzerland.

### Cognitive Evaluations

The used tests were primarily selected to assess the main neuropsychological functions. Unless otherwise stated, tests were taken from “Materials and Norms for the Neuropsychological Diagnostics,” and normal values were available for all tests (20). Tests for verbal and visual short-term memory included the Digit Span Forward and a Block Tapping test. To measure learning and long-term memory, the Rey Auditory Verbal Learning test, the Rey Visual Design Learning test, and the delayed recall of Logical Memory of the Wechsler Memory Scale and the delayed recall (30′) of Rey’s Complex Figure were used. Performance on executive functions were assessed with a Word and Design fluency task, Stroop’s Color-Word test, a Planning (tower) test, and the Kramer’s test, i.e., a Card Sorting test. Tests for measuring different aspects of attention included two subtests (alertness and divided attention) of the computerized test battery of Zimmermann and Fimm (21), the Test des Deux Barrages (assessing focused attention), and an extended German version of part A of the Trail Making test. Visuospatial constructional ability was tested by means of copying the Complex Figure of Rey. A test called Spatial test assessed mental rotation.

Tests without normative data included the Bells test (assessing neglect; cut-off: 6 of maximum 36 omissions); the test of upper limb apraxia (TULIA; cut-off: 194 of maximum 240 points); language tasks assessing comprehension (short version of the token test); naming, reading, and writing (each of these maximum 10 points); and the Cortical Vision Screening test (maximum 38 points) (22).

### Behavioral Evaluation

The FrSBe assesses behavioral changes related to frontal lobe disturbances, which includes apathy, disinhibition, and executive dysfunction that contribute to a total score (23). It is filled out by the patient (“self-report form”) and additionally by a designated significant other or family member (“family form”), retrospectively with regard to the behavioral status before first symptoms of MS (called “before illness”) and with regard to the current status (called “after illness”). The FrSBe was shown to be a sensitive measure of behavioral changes and a predictor of neuropsychological deficits and poor adaptive function in patients with MS. It was validated using a healthy control group and normal values are available (6, 9).

The Beck Depression Inventory (BDI) test to evaluate depressive symptoms (24), the Wuerzburg Fatigue Inventory for Multiple Sclerosis (WEIMuS) test to evaluate fatigue (25), and the FrSBe (23) were performed.

### Statistical Analysis

Statistical analyses were exploratory and were performed using R (version 3.1.2, R Core Team) (26). Analyses were based on all evaluable patients, and missing values were not imputed. Normal values were available for all analyses. Unless otherwise stated, normalized T scores corrected for age, sex, and education were calculated. T scores have a mean of 50 and a SD of 10. With the exception of the FrSBe, T scores ≤35 are pathological, and T scores of 36–40 may be interpreted as borderline impairment. With respect to FrSBe, T scores ≥65 are pathological. T scores of 60–64 may be interpreted as borderline impairment.

The relationship of behavioral changes measured by the FrSBe and disease characteristics (age, gender, education, disease duration, MS type, and treatment) as well as disability (EDSS) was assessed by multivariable analysis of covariance. To analyze the relationship of behavioral changes to the neuropsychological and behavioral evaluations, Spearman rank correlation coefficients were calculated between the FrSBe self- and family-form postillness with all neuropsychological tests, the BDI, and the WEIMuS. The level of significance of two-sided statistical tests was set at *p* = 0.05, and no correction for multiple testing was applied.

To improve sensitivity to the different cognitive domains, additional mean scores of the respective tests for the domains “Verbal memory,” “Non-verbal memory,” “Executive function,” “Attention,” “Visuospatial Abilities/Visual perception,” and “Language” were calculated and correlated to the FrSBe as well (see above, Tables 2 and 4).

The differences in the assessment of behavioral changes by patients and by significant others, the “self-report form” of the FrSBe (filled out by the patient), and the “family form” of the FrSBe (filled out by a designated significant other or family member), with regard to the behavioral status before the first symptoms of MS (called “before illness”) and the current status (called “after illness”), were compared using the Wilcoxon signed-rank test.

## Results

### Patient Population

66 consecutive patients were included. The characteristics of the study population regarding age, gender, education and disease duration, mean EDSS, EDSS distribution, MS type, and current immunomodulatory therapy are shown in Table 1. There were no relevant comorbidities. Non-steroidal anti-inflammatory drugs and antidepressants were the most common additional medical treatments. Patients with higher EDSS scores had more muscle relaxants.

**Table 1:** Patients characteristics (*n* = 66).

Age (years), mean ± SD (range)	43.4 ± 11.9 (21–68)
Gender (female), %	75.8
Education duration (years), mean ± SD (range)	13.2 ± 2 (9–18)
Disease duration (years), mean ± SD (range)	9.3 ± 7.9 (0.5–46.2)
EDSS, mean ± SD (range)	3.0 ± 1.6 (0–7)
EDSS distribution, *n* (%)
0.0–0.5	1 (1.5%)
1.0–1.5	16 (24.2%)
2.0–2.5	19 (28.8%)
3.0–3.5	11 (16.7%)
4.0–4.5	10 (15.2%)
5.0–5.5	2 (3%)
6.0–6.5	6 (9.1%)
7.0	1 (1.5%)
MS type, *n* (%)	
CIS	4 (6%)
RRMS	52 (79%)
SPMS	8 (12%)
PPMS	2 (3%)
Immunomodulatory therapy, *n* (%)	
Interferon beta	23 (34.8%)
Natalizumab	20 (30.3%)
Glatirameracetate	6 (9.1%)
Mitoxantrone	3 (4.6%)
Azathioprine	1 (1.5%)
No therapy	13 (19.7%)

*n, number; EDSS, Expanded Disability Status Scale; CIS, clinically isolated syndrome; RRMS, relapsing-remitting multiple sclerosis; SPMS, secondary progressive multiple sclerosis; PPMS, primary progressive multiple sclerosis*.

The descriptive results of the cognitive and behavioral tests including the prevalence of pathological test results are outlined in Table 2.

**Table 2:** Results of cognitive and behavioral tests (*n* = 66).

Cognitive and behavioral tests	*T* value, unless indicated with^a^, mean ± SD (range)	Prevalence of pathological values, *n* (%)
**Verbal memory**		
Verbal short-term memory	54.37 ± 7.73 (39–72)	0/66 (0%)
Auditory verbal learning and memory test (learning)	45.92 ± 8.31 (25–65)	6/66 (10%)
Auditory verbal learning and memory test (long-term recall)	50.21 ± 10.8 (22–64)	5/66 (7.58%)
Logical Memory of the Wechsler Memory test	52.34 ± 7.53 (37–72)	0/66 (0%)
Mean values of verbal memory tests	50.59 ± 5.83 (30.75–62.75)	

**Non-verbal memory**		
Visual short-term memory	52.75 ± 9.24 (25–77)	3/66 (4.55%)
Figural learning and memory test (learning)	48.27 ± 9.07 (21–65)	4/65 (6.15%)
Figural learning and memory test (long-term recall)	48.68 ± 10.18 (20–64)	5/64 (7.81%)
Recall of Rey-Taylor complex figure test	53.83 ± 8.98 (20–79)	1/66 (1.52%)
Mean values of non-verbal Memory tests	50.89 ± 6.4 (34.25–66.25)	

**Executive functions**		
Word fluency task	47.40 ± 8.68 (31–72)	5/66 (7.58%)
Design fluency task	50.12 ± 7.82 (28–64)	3/66 (4.55%)
Stroop’s color-word test III (mistakes)	49.98 ± 8.51 (32–58)	2/66 (3.03%)
Stroop’s color-word test III (time)	52.66 ± 14.96 (28–79)	7/66 (10.61%)
Planning test (number of moves)	46.73 ± 10.73 (28–65)	11/64 (17.19%)
Planning test (quality total)	49.21 ± 10.91 (31–65)	7/64 (10.94%)
Kramer’s card sorting test	54.51 ± 7.38 (38–62)	0/66 (0%)
Mean values of executive functions tests	50.12 ± 5.42 (38–63.71)	

**Attention**		
Alertness without warning signal	43.6 ± 10.19 (27–84)	11/65 (16.92%)
Alertness with warning signal	41.55 ± 8.12 (24–63)	15/65 (23.08%)
Divided attention	45.07 ± 9.41 (27–62)	12/65 (18.46%)
Test des deux barrages (total per minute)	42.04 ± 7.50 (20–55)	9/63 (14.29%)
Test des deux barrages (omission)	49.30 ± 8.39 (32–67)	2/63 (3.17%)
Test des deux barrages (constancy)	47.15 ± 11.0 (28–76)	8/63 (12.7%)
Modified Trail Making Test Form A	47.66 ± 8.6 (20–60)	4/57 (7.02%)
Mean values of attention tests	44.98 ± 4.96 (31.67–54.57)	

**Visuospatial abilities/visual perception**		
Rey-Taylor complex figure test (copy)	51.84 ± 4.34 (35–53)	4/66 (6.06%)
Spatial test	52.74 ± 7.7 (30–60)	1/66 (1.52%)
Cortical Vision Screening test (maximum 38)	37.81^a^ ± 0.81 (32–38)	0/66 (0%)
Mean values of visuospatial abilities/visual perception tests	47.6 ± 3.41 (32.33–52)	

**Neglect**		
Bells test (omissions; cut-off 6)	0.96^a^ ± 1.24 (0–5)	0/63 (0%)

**Apraxia**		
Apraxia total (TULIA)	216.93^a^ ± 10.87 (193–237)	3/59 (5.08%)

**Language**		
Token test (maximum 10)	10^a^ ± 0 (10)	0/66 (0%)
Naming (maximum 10)	9.96^a^ ± 0.25 (8–10)	0/66 (0%)
Reading (maximum 10)	10^a^ ± 0 (10)	0/64 (0%)
Writing (maximum 10)	9.91^a^ ± 0.52 (6–10)	0/66 (0%)
Mean values of language tests	9.97 ± 0.14 (9–10)	

**Frontal System Behavior Scale**		
***Before***		
Self-rating		
Apathy	48.09 ± 10.26 (30–85)	3/61 (4.91%)
Disinhibition	53.04 ± 10.23 (35–79)	7/61 (11.47%)
Executive dysfunction	50.86 ± 10.61 (33–88)	5/61 (8.47%)
Total	51.24 ± 10.43 (37–95)	7/61 (11.47%)
Family rating		
Apathy	46.55 ± 10.52 (28–72)	4/59 (6.77%)
Disinhibition	48.45 ± 10.66 (28–71)	5/59 (8.19%)
Executive dysfunction	51.84 ± 13.28 (28–97)	8/59 (12.12%)
Total	49.27 ± 11.45 (25–77)	8/59 (12.12%)
***After***		
Self-rating		
Apathy	59.78 ± 15.64 (32–111)	17/61 (27.86%)
Disinhibition	54.42 ± 9.92 (40–79)	14/61 (22.95%)
Executive dysfunction	55.91 ± 12.48 (33–91)	14/61 (22.95%)
Total	58 ± 12.0 (36–90)	15/61 (24.59%)
Family rating		
Apathy	55.08 ± 15.09 (28–93)	11/60 (10.00%)
Disinhibition	54.46 ± 13.76 (28–91)	17/60 (27.86%)
Executive dysfunction	55.95 ± 15.18 (28–93)	20/60 (33.33%)
Total	56.85 ± 15.11 (25–100)	18/60 (30%)

**BDI**	12.34^a^ ± 7.95 (0–36)	
	0–8 (none depressive symptoms)	22/61 (36.06%)
	9–13 (minimal depressive symptoms)	16/61 (26.22%)
	14–19 (mild depressive symptoms)	13/61 (21.31%)
	20–28 (medium depressive symptoms)	7/61 (11.47%)
	>29 (heavily depressive symptoms)	3/61 (4.91%)

**WEIMuS**		
Physically, comparison with HC	61.54 ± 14.81 (39–89)	8/62 (12.90%)
Cognitive, comparison with HC	60.41 ± 13.87 (40–88)	14/62 (22.58%)
Total, comparison with HC	61.11 ± 14.53 (39–91)	10/62 (16.12%)
Physically, comparison to PwMS	43.83 ± 11.43 (26–65)	8/62 (12.90%)
Cognitive, comparison to PwMS	47.62 ± 9.76 (33–68)	11/62 (17.74%)
Total, comparison to PwMS	45.51 ± 11.38 (28–68)	11/62 (17.74%)

*The results of each cognitive or behavioral test are shown and mean values of tests contributing to the cognitive domains “Verbal memory,” “Non-verbal memory,” “Executive function,” “Attention,” “Visuospatial Abilities/Visual perception,” and “Language” were calculated; T scores ≤35 are pathological except for the FrSBe in which T value >65 are pathological*.

*^a^Raw value of scale*.

*n, number, HC, healthy controls; PwMS, patients with multiple sclerosis; BDI, Beck Depression Inventory; WEIMuS, Wuerzburg Fatigue Inventory for Multiple Sclerosis test; FrSBe, Frontal Systems Behavior Scale*.

### Cognitive Test Results

With regard to cognitive functions, patients were largely unimpaired in memory, executive functions, visuospatial abilities/perception, neglect, apraxia, and language (pathological values most often in less than 10% of patients), with the exception of planning abilities. In different aspects of attention, we found deficits in up to 23% of patients (Table 2).

### Behavioral Test Results

**Figure 1:**
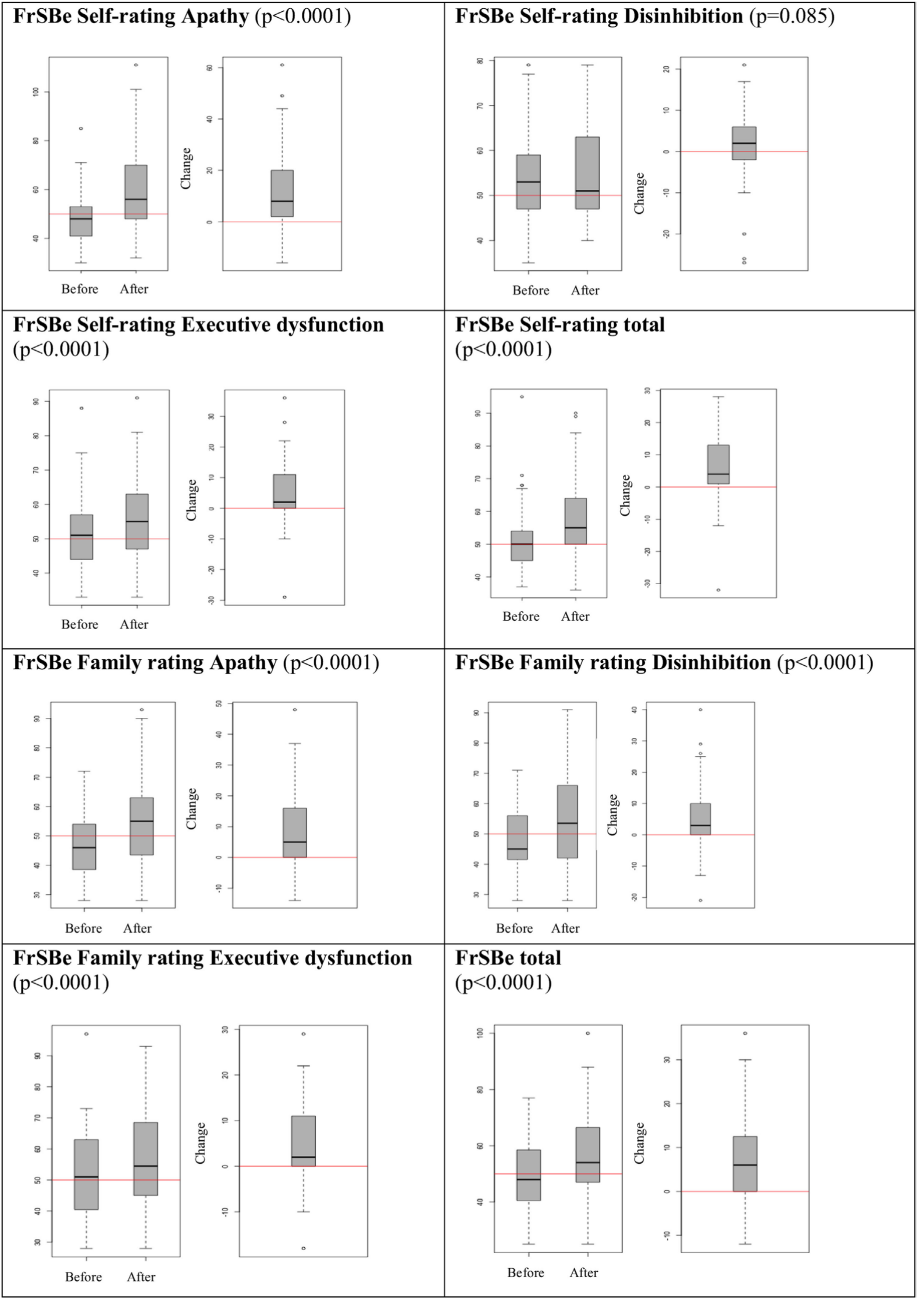
Behavioral changes before vs. after illness.

With regard to the FrSBe, the total score for behavioral self-ratings showed pathological values in 11.47% before and 24.59% after illness. With regard to the behavioral domains of the FrSBe, i.e., apathy, executive dysfunction, or disinhibition, up to 11.47% patients before and up to 27.9% patients after illness showed pathological values in at least one domain. There was a significant increase after illness compared to before illness in all FrSBe scales (all *p* < 0.0001) except for the domain “disinhibition” that showed a non-significant trend (*p* = 0.08) (Figure 1).

Behavioral family rating of the FrSBe showed a pathological total score in 12.12% before illness and 30% after illness and in up to 12.1% before illness and up to 33.3% after illness in one of the examined FrSBe domains (Table 2). There was a significant increase of all FrSBe scales after illness compared to before illness (all *p* < 0.0001) (Figure 1).

In the BDI, 36% of patients did not have depressive symptoms, 59.1% showed minimal to medium depressive symptoms, and 4.9% heavily depressive symptoms (Table 2).

The WEIMuS showed higher rates of cognitive than physical fatigue, with rates of up to 22.58% compared to healthy controls and up to 17.74% compared to MS patients (Table 2).

The TULIA showed three patients with mild apraxia (4.5% of study population) and none with moderate or severe apraxia (normal values taken from the studs by Vanbellingen et al.) (27).

### Comparison of Patients and Significant Others in Rating Behavioral Changes

**Figure 2:**
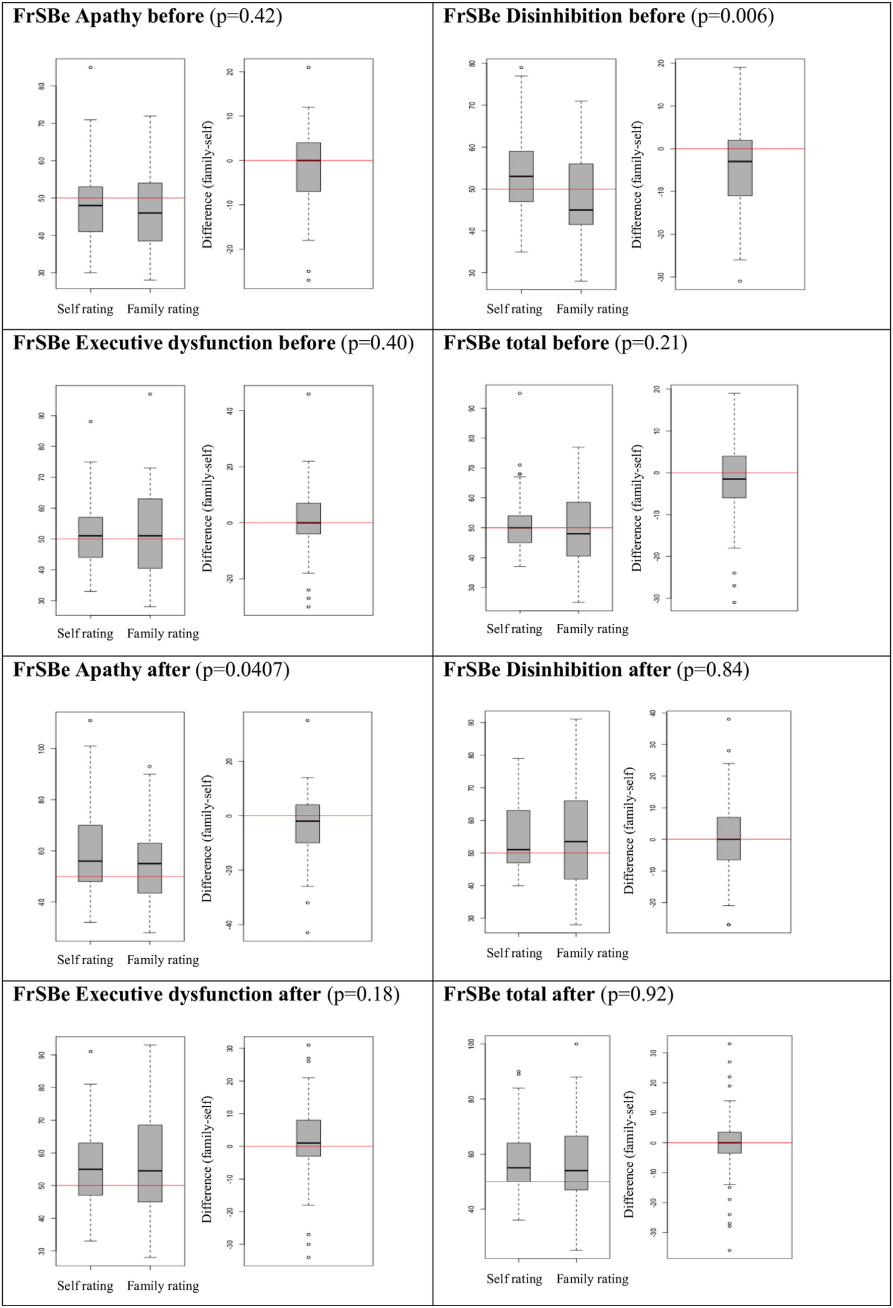
Behavioral changes: self- vs. family rating.

Self-ratings and family ratings of the FrSBe did not show significant differences except for the domains “apathy after illness” and “disinhibition before illness” (*p* = 0.0407 and *p* = 0.0066, respectively), with higher values in the self-ratings (Figure 2).

### Correlation of Behavioral Changes (FrSBe Results) with MS Disease-Specific Characteristics

The correlations of the FrSBe scales with disease-specific aspects are outlined in Table 3. The strongest correlation was found between the family ratings and PPMS, especially in the disinhibition scale. Higher FrSBe test values before illness were indicative for pathological test results after illness.

**Table 3:** Contribution (coefficients) of disease characteristics to behavioral changes.

	FrSBe self-ratings				FrSBe family ratings			
	Apathy	Disinhibition	Executive dysfunction	Total score	Apathy	Disinhibition	Executive dysfunction	Total score
Age	0.11	−0.065	0.18	0.17	0.05	−0.25	−0.06	−0.04
Female vs. male	6.97	7.84^†^	5.81	7.78*	5.77	3.65	5.11	4.8
Education duration (years)	−0.14	0.82	0.32	0.48	−0.8	0.08	0.155	−0.16
Disease duration (years)	−0.34	−0.03	−0.25	−0.205	0.6	0.77^†^	0.3	0.62*
Examination duration (minutes)	0.47	−4.96*	−0.39	−2.0	1.04	1.84	−0.26	0.35
MS type								
CIS vs. RRMS	−7.79	−0.465	−5.095	−5.71	5.97	11.51	1.695	6.15
SPMS vs. RRMS	3.03	−0.94	−2.68	1.355	9.46	−0.465	−4.9	4.78
PPMS vs. RRMS	−7.25	9.775	−7.32	−3.07	24.15^†^	30.88^†^	2.85	24.36*
EDSS	2.72	2.88^†^	2.79	2.7	0.23	1.11	2.71	1.11
Not treated vs. treated	3.97	0.62	2.08	2.26	−6.94	−13.42^†^	−0.3	−6.72

*p < 0.05.

*^†^p < 0.01*.

*EDSS, Expanded Disability Status Scale; CIS, clinically isolated syndrome; RRMS, relapsing-remitting multiple sclerosis; SPMS, secondary progressive multiple sclerosis; PPMS, primary progressive multiple sclerosis; FrSBe, Frontal Systems Behavior Scale*.

### Correlation of Behavioral Changes (FrSBe Results) with Cognitive and Behavioral Tests

The correlations of the FrSBe with all cognitive and behavioral tests are outlined in Table 4. The strongest and most consistent correlation was found between the FrSBe (self- and family rating) and the WEIMuS and BDI. Otherwise, only single significant correlations were found as indicated in Table 4. In addition, the correlation of the FrSBe with a frontal lobe test battery resembling the cognitive domain “Executive function” (= mean values of “Word fluency task,” “Design fluency task,” “Stroop’s color-word test III (mistakes),” “Stroop’s color-word test III (time),” “Planning test (number of moves),” “Planning test (quality total),” and “Kramer’s card sorting test”) showed a significant correlation to Executive dysfunctions (self-rating) of the FrSBe. All other correlations were not significant.

**Table 4:** Correlation between FrSBe and cognitive tests.

Tests	FrSBe self-ratings				FrSBe family ratings			
	Apathy	Disinhibition	Executive dysfunction	Total score	Apathy	Disinhibition	Executive dysfunction	Total score
**Verbal memory**								
Verbal short-term memory	−0.108	−0.04	−0.33^†^	−0.17	0.12	−0.085	−0.14	−0.05
Auditory verbal learning and memory test (learning)	−0.1	0.07	−0.05	−0.005	−0.02	0.05	0.05	0.04
Auditory verbal learning and memory test (long-term recall)	0.09	0.20	0.08	0.17	0.05	0.166	0.1	0.1
Logical Memory of the Wechsler Memory test	−0.1055	−0.04	−0.24	−0.15	−0.25	−0.23	−0.25	−0.28*
Mean value of verbal memory tests	−0.08	0.07	−0.18	−0.03	−0.02	−0.03	−0.08	−0.05

**Non-verbal memory**								
Visual short-term memory	−0.086	0.035	−0.17	−0.16	−0.08	−0.1	−0.21	−0.14
Figural learning and memory test (learning)	−0.023	0.285*	0.04	0.09	−0.18	0.04	−0.03	−0.05
Figural learning and memory test (long-term recall)	0.033	0.23	0.05	0.08	−0.20	0.02	−0.05	−0.085
Recall of Rey-Taylor complex figure test	0.14	0.05	0.08	0.12	−0.009	0.08	0.04	0.02
Mean value of non-verbal memory tests	0.03	0.16	−0.01	0.01	−0.13	−0.02	−0.07	−0.09

**Executive functions**								
Word fluency task	−0.21	−0.06	−0.23	−0.22	−0.09	0.008	−0.14	−0.085
Design fluency task	−0.06	−0.0007	−0.17	−0.13	−0.28*	−0.21	−0.13	−0.23
Stroop’s color-word test III (mistakes)	−0.05	0.01	−0.05	−0.03	0.11	0.21	0.15	0.1
Stroop’s color-word test III (time)	−0.19	−0.19	−0.23	−0.19	−0.04	−0.13	−0.09	−0.12
Planning test (number of moves)	−0.06	−0.135	−0.1	−0.12	−0.04	−0.21	−0.15	−0.14
Planning test (quality total)	−0.02	−0.03	−0.03	−0.05	−0.003	−0.2	−0.14	−0.11
Kramer’s card sorting test	0.11	0.09	−0.14	−0.02	−0.01	−0.02	−0.02	−0.025
Mean value of Executive functions tests	−0.17	−0.15	−0.27*	−0.23	−0.09	−0.18	−0.19	−0.18

**Attention**								
Alertness without warning signal	−0.25	0.13	−0.19	−0.175	−0.22	−0.19	−0.23	−0.24
Alertness with warning signal	−0.09	0.07	−0.1	−0.09	−0.19	−0.13	−0.15	−0.18
Devided attention	0.18	0.21	0.19	0.25*	0.01	−0.12	0.03	−0.015
Test des deux barrages (total per minute)	−0.03	0.2	−0.21	−0.001	−0.06	−0.035	−0.105	−0.07
Test des deux barrages (omission)	−0.0026	0.34^†^	0.04	0.109	0.035	0.08	−0.002	0.05
Test des deux barrages (constancy)	−0.19	−0.086	−0.24	−0.19	−0.14	−0.17	−0.236	−0.2
Modificated Trail Making test Form A	−0.19	0.41^†^	−0.11	0.006	−0.12	−0.136	−0.15	−0.13
Mean value of attention tests	−0.21	0.22	−0.25	−0.12	−0.21	−0.17	−0.24	−0.23

**Visuospatial abilities/visual perception tests**								
Rey-Taylor complex figure test (copy)	0.04	0.116	0.23	0.19	0.11	0.06	0.19	0.155
Spatial test	−0.09	−0.23	−0.11	−0.115	−0.05	−0.04	0.03	0.015
Cortical Vision Screening test (maximum 38)	−0.01	0.06	−0.18	−0.12	−0.03	0.1	−0.02	−0.004
Mean values of visuospatial abilities/visual perception tests	−0.05	−0.21	−0.07	−0.09	0.03	0.04	0.04	0.07

**Neglect**								
Bells test (omissions; cut-off 6)	0.15	−0.15	0.06	0.056	0.03	−0.02	0.13	0.09

**Apraxia**								
Apraxia total	−0.15	0.055	−0.136	−0.11	−0.13	−0.11	−0.1	−0.13

**Language**								
Token test (maximum 10)	NA	NA	NA	NA	NA	NA	NA	NA
Naming (maximum 10)	−0.16	−0.13	−0.14	−0.17	−0.095	−0.09	−0.06	−0.08
Reading (maximum 10)	NA	NA	NA	NA	NA	NA	NA	NA
Writing (maximum 10)	−0.04	−0.02	−0.13	−0.13	−0.13	−0.12	−0.17	−0.185
Mean values of Language tests	−0.11	−0.08	−0.17	−0.18	−0.15	−0.16	−0.17	−0.19

**Beck Depression Inventory**	0.58^†^	0.38^†^	0.57^†^	0.64^†^	0.42^†^	0.29*	0.42^†^	0.45^†^

**WEIMus**								
WEIMuS physically, comparison with healthy controls	0.71^†^	0.246	0.59^†^	0.7^†^	0.45^†^	0.35^†^	0.48^†^	0.51^†^
WEIMuS cognitive, comparison with healthy controls	0.76^†^	0.266*	0.63^†^	0.765^†^	0.5^†^	0.335^†^	0.47^†^	0.51^†^
WEIMuS total, comparison with healthy controls	0.76^†^	0.276*	0.63^†^	0.76^†^	0.47^†^	0.34^†^	0.49^†^	0.51^†^
WEIMuS physically, comparison with MS patients	0.7^†^	0.23	0.6^†^	0.7^†^	0.45^†^	0.32*	0.49^†^	0.5^†^
WEIMuS cognitive, comparison with MS patients	0.76^†^	0.29*	0.65^†^	0.79^†^	0.48^†^	0.35^†^	0.48^†^	0.5^†^
total, comparison with MS patients	0.74^†^	0.27*	0.63^†^	0.75^†^	0.47^†^	0.34^†^	0.48^†^	0.5^†^

*p < 0.05

*^†^p < 0.01*.

*WEIMuS, Wuerzburg Fatigue Inventory for Multiple Sclerosis; MS, multiple sclerosis; FrSBe, Frontal Systems Behavior Scale*.

### Multivariable Linear Models of the FrSBe Including BDI, WEIMuS, and Frontal Lobe Tests

We used multivariable linear models to investigate the contribution of measures of depressive symptoms and/or fatigue concurrently with frontal lobe tests (Kramer test correct ones, Word fluency, Design fluency, and Planning test moves) to the current self-rating FrSBe scores. Data indicate that fatigue and depressive symptoms were in most cases significant predictors for FrSBe test results (only for WEIMuS did not predict disinhibition), and frontal lobe tests were not.

## Discussion

Up to one third of patients of our cohort showed behavioral changes in at least one domain (apathy, disinhibition, and executive dysfunction) or in the total score of the FrSBe. Apart from this, patients were rather mildly affected, especially with regard to cognitive functioning. Less than 10% of patients showed pathological values in the performed cognitive tests with the exception of planning abilities and different aspects of attention that showed deficits in up to 23% of patients (Table 2).

There were no consistent correlations of behavioral changes with the EDSS, gender, disease duration, and MS type. Only PPMS consistently predicted the occurrence of behavioral symptoms in the family ratings, a finding that is, however, difficult to interpret due to the small PPMS sample (Table 3).

Cognitive impairments and especially frontal lobe dysfunction did not consistently predict behavioral changes as well. Only the mean value of the executive dysfunctions test battery, resembling a cognitive frontal lobe test battery, was significantly correlated with self-rating of executive dysfunctions of the FrSBe (Table 4).

Taken together, these results indicate that behavioral changes are present in a relevant proportion of otherwise mildly affected MS patients especially with regard to cognitive functioning. In these patients, behavioral changes occur largely independent of disease characteristics, physical disability, and cognitive impairments. Only depressive symptoms and fatigue were strongly associated with behavioral changes, a finding that was well established from previous studies (9, 28). Therefore, the existence of behavioral changes in MS patients cannot be necessarily anticipated from the performed clinical outcome measurements. For this reason, behavioral changes should be specifically tested, which is of importance because behavioral changes are relevant impairments that negatively influence the daily life of patients and working ability (6). In addition, alternative diagnostic tools to predict or diagnose behavioral changes would be of great interest. Magnetic resonance imaging (MRI) studies, especially cortical thickness and deep gray matter volumetry, have been shown to correlate with cognitive impairment and behavioral changes such as depression (29, 30). Therefore, MRI studies were performed in all patients in this study including the analysis of cortical thickness and cortical lesions that will be assessed in 33 different anatomical brain areas using a T1-modified driven equilibrium Fourier transform sequence and double inversion recovery sequence, respectively. This currently ongoing analysis aims to examine potential correlations between cortical pathologies and behavioral changes (beside depression and fatigue).

In contrast to our study, Chiaravalloti and DeLuca found significant correlations between cognitive impairments and behavioral changes also analyzed by the FrSBe (9). This is probably due to a presumably more cognitive impaired patient cohort in the Chiaravalloti study compared to our cohort. However, an exact comparison of both cohorts is not possible due to the different assessments of disability and cognitive functioning in both studies. Overall, our study therefore adds important information on behavioral changes in physically and cognitively mildly affected MS patients, which broadens the knowledge about behavioral changes in MS patients.

Behavioral changes were reported more often after illness compared to before illness (Figure 1) and self- and family ratings of the current behavioral status were equal in most scores (Figure 2), which is in line with the study performed by Chiaravalloti and DeLuca (9). These findings are of interest because self- and family ratings of behavioral changes as well as cognitive impairments in MS patients showed large discrepancies in previous studies that did not use the FrSBe for the evaluation of behavioral changes (13, 14). In addition, self- and family ratings of behavioral changes usually differ substantially in other brain diseases such as traumatic brain injuries or brain tumors (15–17).

These results indicate suggest that the FrSBe is suitable for the reliable evaluation of behavioral changes asking both, the MS patients or family members and can be used in the daily routine.

### Study Limitations

The study was performed in a mildly affected MS patient cohort especially with regard to cognitive functioning, and therefore, conclusions for moderately to severely affected patients cannot be made. The tests used in this study are validated and existing normative values were compared to the results of our study population. However, we did not include an own control group and did therefore not account for differences, which might be population specific to our group. Validated subjectively reported outcome measurements, answered by patients and family members, were used in the study. Furthermore, the number of CIS and PPMS patients was low rendering any conclusions impossible.

## Conclusion

Behavioral changes are common even in otherwise mildly affected MS patients with up to one third of patients being affected. They occur largely independently of disease characteristics, physical disability, and cognitive functioning but correlate with both fatigue and depressive symptoms. Therefore, behavioral changes can not necessarily be anticipated in MS patients and should be specifically evaluated to obtain a more comprehensive picture of patients’ symptoms. By using the FrSBe, patients and family members can be asked with regard to behavioral changes.

## Ethics Statement

This study was carried out in accordance with the Declaration of Helsinki. The study was approved by the ethics committee of the canton of Bern, Switzerland. All subjects gave written informed consent prior to study entry.

## Author Contributions

MH organized, designed and managed the study. She did data collection, data analysis, and wrote the paper. Additionally she recruited patients. SK did all cogntivie tests, did patient management, did data analysis, and helped writing the paper. KG helped designing the study, did data analysis, and helped writing the paper. He additionally helped organizing the study. CB did the main part oft the statistical analysis being a professional statistician. SB helped designing the study, did data analysis, and helped writing the paper. VB did patient recruiting, data analysis, and helped writing the paper. HM helped designing the study, did data analysis, and helped writing the paper. He additionally helped organizing the study. RM helped designing the study, did data analysis, and helped writing the paper. He additionally helped organizing the study. RV organized and designed the study and recruited patients. He helped writing the paper. CK organized, designed, and managed the study. He organized funding, did data collection, data analysis, and wrote the paper.

## Conflict of Interest Statement

The authors declare that the research was conducted in the absence of any commercial or financial relationships that could be construed as a potential conflict of interest.
